# Knowledge, attitudes, and practices (KAP) regarding occupational protection among orthopedic theatre nurses using orthopedic power tools (OPTs): A cross-sectional study

**DOI:** 10.1371/journal.pone.0349690

**Published:** 2026-05-15

**Authors:** Hang Li, Zhao-Li Guo, Tao Chen, Wen-Qian Wang, Zheng Gao

**Affiliations:** 1 Operating Room, Fenyang Hospital of Shanxi Province, Shanxi, Fenyang, China; 2 Nursing Department, Fenyang Hospital of Shanxi Province, Shanxi, Fenyang, China; 3 Medical Records Room, Fenyang Hospital of Shanxi Province, Shanxi, Fenyang, China; Nippon Medical School, JAPAN

## Abstract

**Introduction:**

The objective of this study was to explore the factors associated with the knowledge, attitudes, and practices (KAP) of orthopedic theatre nurses with regard to occupational protection when orthopedic power tools (OPTs) are used to provide a reference for ensuring the occupational safety of nurses working in orthopedic surgery rooms.

**Methods:**

This cross-sectional survey involved 272 orthopedic theatre nurses across eight tertiary hospitals in Shanxi Province, China, between September and December 2024. An occupational protection assessment tool that was self-developed and included 3 dimensions (Knowledge, Attitude, and Practice), containing a total of 45 items, was utilized among operating theatre nurses with OPTs.

**Results:**

The median as the cutoff, 52.9% (144/272) of respondents had an adequate Knowledge score, while 47.1% (128/272) had a good score; 54.0% (147/272) of the respondents had a positive Attitude score, and 46.0% (125/272) had a negative attitude; 53.7% (146/272) of the participants demonstrated good performance in the Practice score, whereas 46.3% (126/272) showed poor performance. Multivariable regression analysis revealed that the knowledge scores of those with a bachelor’s degree (aOR=7.040; 95% CI: 3.204–15.466; p < 0.001), senior professional title (aOR=5.216; 95% CI: 1.228–22.155; p = 0.025), occupational protection training frequency less than 6 months (aOR=10.085; 95% CI: 4.146–24.533; p < 0.001) or 6–12 months (aOR=6.550; 95% CI: 3.262–13.153; p < 0.001) and actively consulting the latest professional protection knowledge (aOR=2.652; 95% CI: 1.426–4.931; p = 0.002) were higher and statistically significant. Senior title (aOR=5.226; 95% CI: 1.409–19.387; p = 0.013), occupational exposure (aOR=2.770; 95% CI: 1.484–5.169; p = 0.001) and Knowledge scores (aOR=2.663; 95% CI: 1.581–4,448; p < 0.001) yielded attitude scores that were higher and statistically significant. Attitude scores (aOR=3.683; 95% CI: 2.106–6.441; p < 0.001), actively consulting the latest professional protection knowledge (aOR=2.962; 95% CI: 1.686–5.201; p = 0.001), and higher Knowledge scores (aOR=2.255; 95% CI: 1.294–3.930; p = 0.004) were independently associated with better practice scores.

**Conclusion:**

Nurses in the orthopedic operating room had a positive attitude toward the occupational protection of using power tools, but their knowledge was insufficient, and their practice behavior was poor. Normative guidelines for occupational protection in the workplace of an orthopedic theatre and a training and evaluation system should be developed. Moreover, future measures should focus on education and training for occupational protection. Additionally, developing and designing occupational protective equipment that is more convenient for orthopedic surgery is important.

**Reporting method:**

This research was designed as a cross-sectional survey study that was conducted in strict accordance with the Strengthening the Reporting of Observational Studies in Epidemiology (STROBE) guidelines.

## Introduction

Occupational exposure of medical staff involves situations in which medical staff may be exposed to toxic or harmful substances or pathogens in the process of diagnosis and treatment, nursing and related work, which may cause potential harm to health [1]. Orthopedic theatre nurses are very susceptible to occupational exposure. In addition to needlestick injuries, anesthesia exhaust inhalation hazards, psoas muscle strain and other occupational hazards, occupational injuries such as sharp cutting injury, blood and body fluid splashing, virus aerosol transmission, and noise-related hearing damage during the use of power tools easily occur among nurses, particularly in orthopedic theatres [2–5]. Orthopedic power tools (OPTs) refer to medical devices that provide power through electric or pneumatic systems to help doctors cut, drill, and grind bones [6]. These tools usually include electric handles, drills, screws and hole openers. Orthopedic power tools have become indispensable in orthopedic surgery, and they play key roles in various surgeries, such as fracture fixation and joint replacement.

Operating room nurses play a critical role in orthopedic surgery. During operations, they frequently experience glove punctures and accidental finger pricks [7]. Owing to the use of heavy and sharp power instruments in orthopedic surgery, blood spatter and aerosol atomization are the second most common modes of occupational exposure for nurses in the orthopedic theatre after sharp instrument injuries [8,9]. Endo et al. [10] conducted a survey among operating room staff and reported that the incidence of blood spattering during orthopedic surgery was 60.0% (90/150) while the incidence rate of hand-washing nurses was 46.0% (92/200). In addition, aerosols are easily generated during many orthopedic procedures, increasing the risk of bacterial or viral infection for the surgical team [11], and studies have shown that aerosols generated by high-speed cutters can spread over an area of up to 6 × 8 m, which takes up the entire operating room during hip joint revision surgery [5,12]. Furthermore, noise-induced hearing loss (NIHL) is an underreported problem in orthopedic surgery. An orthopedic electric drill, an orthopedic saw, a negative pressure suction device and hammering during surgery can generate noise levels up to 145 dB [13]. In the orthopedic operating room environment, excessive noise may have adverse effects on patients, surgeons and their team members. The prevalence of hearing loss is significantly greater among joint replacement surgeons than among nonsurgical surgeons [14]. At present, research on hearing protection among nurses in the orthopedic operating room is insufficient, and we do not know about the implementation of hearing protection among nurses in the orthopedic operating room.

In regard to the use of orthopedic power tools, the literature focuses on either doctors’ operational techniques or reports only single-factor monitoring of sharp instrument injuries, aerosols, noise, and blood-borne exposures. Integrated measurement and mechanism verification of the protective knowledge, beliefs, and actual behaviors of orthopedic operation room nurses are lacking. This study was the first to introduce the KAP framework to the use of orthopedic power tools and to develop and validate the “Orthopedic Theatre Nurses’ Knowledge–Attitude–Practice Scale for Power Tool Occupational Protection”. The mainstream academic literature in this interdisciplinary field has been supplemented and provided a replicable tool and theoretical model for subsequent intervention studies.

This study aims to comprehensively assess orthopedic operating room nurses’ knowledge, attitudes, and practices and associated factors regarding occupational protection when orthopedic power tools (OPTs) are used. This study provides a reference for further improving the protection strategies of orthopedic operating room nurses and ensuring their occupational safety.

## Materials and methods

### Ethical consideration

This study was approved by the Ethics Committee of Fenyang Hospital of Shanxi Province in China (approval number: 2024013). All the participants were fully informed about the purpose and procedures of the study while ensuring that their personal information was kept strictly confidential. In addition, participants were clearly informed of their right to withdraw from the study at any time without suffering any adverse effects as a result. When the questionnaires were distributed, the content of the informed consent form was already conveyed to the participants in electronic text form. From September 1st to December 31st, 2024, this study selected orthopedic theatre nurses from eight tertiary hospitals in Shanxi Province as study participants.

### Study design and participants

A cross-sectional design was used in this study to assess occupational protection practices among orthopedic theatre nurses using OPTs. The included participants satisfied the following criteria: (1) had obtained a nurse’s license, (2) had worked in the orthopedic operation room for more than 6 months, and (3) were willing to participate in the study. Nursing students and transferred nurses during the training period were excluded. In this study, a census was used to conduct a questionnaire survey among 290 registered nurses in an orthopedic theatre who agreed to participate in the study. All exposure and behavioral measures were obtained by self-report, which may be subject to recall and social desirability bias.

### Sample size

The sample size was calculated by the following formula: [n = (z^2^ p (1-p))/d^2^], where z = 1.96 for the 95% confidence level and the 6% allowable error range (d = 0.06). In accordance with previous research findings, the proportion of the expected population was set at 50%. On the basis of the aforementioned formula, the minimum required sample size was ultimately determined to be 267.

### Procedures

#### General information questionnaire.

A nine-item questionnaire covering sociodemographic and occupational characteristics was used in this study. It specifically collected information on sex, age, education level, length of work experience, job title, willingness to participate in training, frequency of training in medical institutions, whether the respondent actively sought the latest professional protection knowledge and history of occupational exposure. Furthermore, self-designed questionnaires concerning knowledge, attitudes and practices regarding occupational protection among operating theatre nurses using OPTs were designed on the basis of the practice of occupational protection of nurses in orthopedic theatres [2–5]. Six experts from the orthopedic surgery, nursing, nosocomial infection and statistical methodology fields were invited to guide and review the design and production of the questionnaire. The experts satisfied the following criteria: (1) had a bachelor’s degree or above, (2) had a senior professional title, and (3) had more than ten years of work experience in the respective field. After two rounds of expert consultation, the Kendall's W coefficients were 0.417 and 0.556, respectively, both of which reached statistical significance (P < 0.05). The content validity indices (CVIs) of knowledge, attitude and practice were 0.901, 0.922 and 0.933, respectively. Duplicate items were merged, ambiguous items were revised (such as the description of hearing protection content), and a new item was added (I know the principles of management and reporting procedures after occupational exposure).

The stability of the final scales was tested using internal consistency reliability and split-half reliability. The Cronbach's α coefficients for the knowledge, attitude, and practice dimensions of the scale were 0.832, 0.821, and 0.767, respectively. Furthermore, the odd-even split-half method was employed for testing. The correlation coefficients of the two halves of the scores for the knowledge, attitude, and practice dimension scales were 0.828, 0.764, and 0.732, respectively; the Spearman–Brown corrected coefficients were 0.906, 0.867, and 0.846, respectively; and the Guttman split-half reliability was 0.905, 0.866 and 0.843, respectively. The above data indicate that these scales have relatively high internal consistency reliability.

To verify the construct validity of the scale, the degree of fit between the model and the observed data was tested by using confirmatory factor analysis (CFA) in AMOS 23.0 software. The model fitting results are as follows: Knowledge dimension χ²/df = 3.509, CFI = 0.845, TLI = 0.806, RMSEA = 0.096; Attitude dimension χ²/df = 3.538, CFI = 0.877, TLI = 0.848, RMSEA = 0.097; and Practice dimension χ²/df = 4.028, CFI = 0.812, TLI = 0.768, RMSEA = 0.106. The standardized loadings of all the items were essentially greater than 0.50 and were significant at the P < 0.05 level. The AVE values were generally approximately 0.50, and the CR values of most factors were greater than 0.70, indicating relatively good convergent validity. Additionally, the square root of the AVE was greater than the correlation coefficient between factors, supporting discriminant validity. In conclusion, the scale of this study demonstrates relatively good structural validity.

The KAP questionnaire was divided into three parts: knowledge, attitude and practice. Each part contained 15 items, and a 5-point Likert scale was used to score the items. (1) The knowledge items addressed protective measures against sharp instrument injuries, high-risk procedures generating harmful aerosols, and blood spatter and noise hazards in orthopedic surgery. Each item was scored on a scale of 1–5, where 1 indicated Not Clear at All and 5 indicated Completely Clear, yielding a total score range of 15–75. (2) The total score of the Attitude dimension items ranged from 15 to 75, with 1 indicating Completely Disagree and 5 indicating Completely Agree, covering the willingness to adopt occupational protection in orthopedic surgery. Reverse scoring was applied for Items 2, 6, and 13: “I think wearing protective gear is a bit of a storm in a teacup”, “It is not necessary to wear double gloves during orthopedic surgery if patients are not suffering from infectious diseases”, and “I think hearing protection for medical staff in orthopedic surgery is of little significance”. (3) The Practice dimension items were also scored on a 5-point scale, with 1 indicating Never and 5 indicating Always, and the total score of this dimension ranged from 15 to 75, covering aspects about taking specific protective measures (including sharp injury, body fluid and blood splashes, aerosols, and NIHL protection). Reverse scoring was used for Items 9 and 14; P9: “I will hand the power device directly to the surgeon because of time constraints or the surgeon’s urging”; P14: “I don't have much time to learn about occupational protection in the operating room”.

Owing to the lack of recognized cutoff values, the median values were used to define adequate (score ≥ median) and inadequate (<median) levels of Knowledge, Attitude, and Practice. In sensitivity analyses, we examined the direction and significance of the effects at the P25, median, and P75 cutoff points. The direction and significance of the associations essentially remained unchanged. Moreover, the continuous outcome model (linear regression) yielded concordant results, confirming that the direction of association was not an artifact of dichotomization. In addition, bootstrap validation (1000 resamples) showed that the median-split OR was stable. In conclusion, the findings obtained using the median as the cutoff point are robust.

### Data collection

Data collection for this study was accomplished using JSJ (JinShuJu, an online form-building and information collection tool), a web-based platform (https://jinshuju.net/) for collecting, analyzing, and managing data. An online questionnaire was used to ensure that each question was answered and none was missed. It took approximately 20 minutes for participants to complete the questionnaire. After obtaining the participants’ consent, we distributed the questionnaires on the JSJ network platform.

The staff who distributed the questionnaires received uniform guidance and training. The training clarified the study’s objectives and importance and highlighted the essential points to be observed during the distribution process. From September to December 2024, the data of nurses in the orthopedic operating rooms of eight tertiary hospitals in Shanxi Province were collected and anonymized.

### Statistical analysis

Statistical analysis was conducted using SPSS software version 26.0 developed by IBM Corporation in Armonk, New York, USA. The mean and standard deviation were used to represent normally distributed data, and the median, 25th percentile, and 75th percentile were used to represent data that were not normally distributed. Count data with different demographic characteristics were represented as N (%).

For normally distributed continuous variables, the KAP (Knowledge, Attitude, and Practice) scores of samples with different demographic characteristics were compared by applying the independent sample t test and analysis of variance (ANOVA) methods; for nonnormally distributed variables, Wilcoxon–Mann–Whitney tests (two-group comparisons) and Kruskal‒Wallis analysis (three or more group comparisons) of variance were applied to identify the factors that might have an impact on KAP scores. Correlation analysis was conducted on the scores of each dimension of KAP, Pearson’s correlation coefficient was used for the correlation analysis of normally distributed data, and Spearman’s correlation coefficient was used for the correlation analysis of nonnormally distributed data.

Bivariable and multivariable logistic regression analyses were conducted with the scores of each dimension as the dependent variable. The variables with statistical significance (P < 0.05) in the single-factor analysis were included in the multifactor logistic regression analysis. P values <0.05 were considered to indicate statistical significance.

## Results

### Characteristics of the participants

Among the 290 questionnaires collected, 272 were valid, resulting in an effective response rate of 93.79%. All participants were licensed nurses who had worked in an orthopedic theatre for more than 6 months.

In this study, the participants were generally well educated, with 70.6% of the participants having bachelor’s degrees. More than half (51.8%) of the participants had worked in the operating room for more than 10 years. However, the professional title distribution was relatively basic, with nearly half of the participants (47.1%) having junior professional titles. In addition, 242 participants, accounting for 89% of the total number, expressed a willingness to participate in orthopedic surgery-related occupational protection training, but 134 (49.3%) participants reported that they had never received occupational protection training related to orthopedic power tools. The characteristics of the participants are presented in detail in Table 1.

**Table 1:** KAP scores of orthopedic theatre nurses related to occupational protection with differing characteristics (n = 272).

Characteristics	N(%)	Knowledge		Attitude		Practice	
		Median (25^th^ percentile, 75^th^ percentile	P	Median (25^th^ percentile, 75^th^ percentile)	P	Median (25^th^ percentile, 75^th^ percentile)	P
**Gender**			0.653		0.493		0.526
Male	59 (21.7)	47 (42,50)		59 (54,62)		48 (40,55)	
Female	213 (78.3)	47 (42,54)		59 (54,65)		48 (42.5,55)	
**Age (years)**			**<0.001**		**<0.001**		**0.005**
<30	81 (29.8)	45 (40,49)		58 (52,63.5)		47 (41.5,54.5)	
≥30 to <40	132 (48.5)	47 (42.25,53)		58.5 (54,64)		48 (41.25,54)	
≥40	59 (21.7)	50 (45,59)		62 (58,68)		51 (46,58)	
**Education level**			**<0.001**		**<0.001**		0.335
College degree	80(29.4)	41.5 (38,46.75)		56.5 (52,61.75)		46 (41,54)	
Bachelor’s degree	192 (70.6)	49 (45,55)		60 (55,65)		49 (42.25,55)	
**Years employed in operation room**			**0.010**		0.157		0.099
<5	41 (15.1)	44 (39,49)		57 (52,63)		46 (40.5,53)	
≥5 to <10	90 (33.1)	48 (43,53)		58.5 (53,65)		48 (41,56)	
≥10	141 (51.8)	48 (42.5,54)		60 (55,65)		49 (43,55)	
**Professional title**			**<0.001**		**<0.001**		**0.003**
Junior	128 (47.1)	45.5 (41,49.75)		58 (52,64)		47 (41,54)	
Intermediate	121 (44.5)	48 (43,53.5)		59 (54,64)		48 (42.5,54)	
Senior	23 (8.5)	51 (50,60)		66 (61,70)		55 (50,58)	
**Occupational protection training willingness**			**0.019**		0.186		0.960
Yes	242 (89.0)	48 (43,53)		59 (54,65)		49.5 (41.75,56)	
No	30 (11.0)	42 (36.75,51.75)		59 (48,64.25)		48 (42,55)	
**Occupational protection training Frequency**			**<0.001**		**<0.001**		**<0.001**
Never	134 (49.3)	43 (40,47)		57 (51.75,62)		43.5 (39,54)	
Every 6–12 months	81 (29.8)	50 (46,55.5)		60 (55,67)		49 (45,54)	
Less than 6 months	57 (21.0)	54 (49.5,61)		61 (58,66)		53 (47,57)	
**Whether actively consulting the latest professional protection knowledge**			**<0.001**		**<0.001**		**<0.001**
Yes	131 (48.2)	50 (46,57)		59 (55,65)		51 (45,57)	
No	141 (51.8)	45 (40,50)		59 (52,64)		46 (40,52)	
**History of occupational exposure**			0.146		**<0.001**		**0.039**
Yes	68 (25)	49 (42,54)		62 (58,66)		50 (44,56)	
No	204 (75)	46 (42,51.75)		58 (53,63.75)		48 (42,54)	

### The distribution of KAP scores for occupational protection when OPTs are used

The median Knowledge score was 47 (with an interquartile range of 42 to 52.7), with the highest score being 73 and the lowest being 28. With a median score of 47 as the cutoff, those with an adequate score of Knowledge accounted for 52.9% (144/272), whereas those with an inadequate score of Knowledge accounted for 47.1% (128/272). Participants had the highest awareness rate of “preoperative screening of blood-borne pathogens” (84.6%), followed by the postexposure disposal process (72.1%). However, awareness of the facts that “electric drill noise can reach 145 dB” and “ >85 dB requires hearing protection” was clearly insufficient, with only approximately 35.7% and 33.8% of respondents answering correctly, respectively. More than half of the respondents (50.7%) did not recognize that masks could protect the cheeks of newly shaved men; (details are provided in Table 2). These findings suggest that while nurses in the orthopedic operating room have a good understanding of blood source protection, noise hazards and facial protection remain significant gaps in knowledge.

**Table 2:** Knowledge dimension score distribution.

	Completely clear (5)	Basically clear (4)	Partly clear (3)	Not very clear (2)	Not clear at all (1)
K1: Patients should be screened for anti-HIV, anti-HCV, HBsAg and syphilis antibodies before operation	134 (49.3)	96 (35.3)	38 (14.0)	4 (1.5)	0 (0)
K2: In close contact with blood and irrigating fluid, power instruments in orthopedic surgery will lead to atomization and aerosol formation	33 (12.1)	78 (28.7)	89 (32.7)	58 (21.3)	14 (5.1)
K3: The aerosol cloud generated during orthopedic surgery can be contaminated by microorganisms such as viruses, bacteria and fungi	27 (9.9)	78 (28.7)	83 (30.5)	69 (25.4)	15 (5.5)
K4: I am aware of the high-risk procedures for aerosol generation in orthopedic surgery	14 (5.1)	66 (24.3)	101 (37.1)	78 (28.7)	13 (4.8)
K5: Conventional surgical masks do not protect against procedures that produce high-risk aerosols (N95 or FFP2 masks are preferred)	26 (9.6)	94 (34.6)	77 (28.3)	48 (17.6)	27 (9.9)
K6: In order to prevent bone debris, blood and body fluids from splashing, the personnel involved in the operation should wear appropriate personal protective equipment	34 (12.5)	82 (30.1)	121 (44.5)	30 (11.0)	5 (1.8)
K7: Use goggles to protect the conjunctiva from contamination	74 (27.2)	106 (39.0)	70 (25.7)	20 (7.4)	2 (0.7)
K8: To effectively protect the newly shaved cheeks and neck area, it is recommended that male health care providers wear a face mask	14 (5.1)	59 (21.7)	61 (22.4)	91 (33.5)	47 (17.3)
K9: The electric drill should be equipped with a protective sleeve to prevent blood splashing and bit breakage	29 (10.7)	58 (21.3)	117(43.0)	49 (18.0)	19 (7.0)
K10: The use of power equipment can easily cause wear to the skin of the hands, and wearing double gloves can reduce the risk of penetration of the inner gloves by 6 times	26 (9.6)	51 (18.8)	129 (47.4)	53 (19.5)	13 (4.8)
K11: Ensure that high-risk power instruments or sharp tools pass through a transition area	26 (9.6)	77 (28.3)	121 (44.5)	39 (14.3)	9 (3.3)
K12: Use instruments or auxiliary tools to install or remove power tools	41 (15.1)	67 (24.6)	109 (40.1)	48 (17.6)	7 (2.6)
K13: Operations such as electric bone drills, cutting saws, and hammering can generate up to 145 dB of noise	9 (3.3)	42 (15.4)	46 (16.9)	117 (43.0)	58 (21.3)
K14: When the intraoperative noise level is greater than 85 dB, hearing protection is recommended for medical staff	7 (2.6)	44 (16.2)	38 (14.0)	118 (43.4)	65 (23.9)
K15: I know the principles of management and reporting procedures after occupational exposure	59 (21.7)	137 (50.4)	51 (18.8)	21 (7.7)	4 (1.5)

The median Attitude score was 59 (54,65), with a minimum score of 40 and a maximum score of 75. With a median score of 59 as the cutoff, 54.0% (147/272) of the respondents had a positive Attitude score, while 46.0% (125/272) had a negative Attitude score. Nurses generally highly agree with the four core protective concepts “mastering the patient's infection history before surgery, standardizing the placement of sharp objects, receiving continuous training, and having institutional policies in place for protection” (support rate 89%−98%). However, approximately 40% of the respondents still reported that wearing protective equipment is “troublesome”, and more than one-third of participants believe that “operating on patients without infection can avoid double-layer gloves”, suggesting that convenience and the indication for double-layer gloves are the key points for the next behavioral intervention; (details are provided in Table 3).

**Table 3:** Attitude dimension score distribution.

	Completely agree (5)	Basically agree (4)	Moderate (3)	Disagree (2)	Completely disagree (1)
A1: It is important to fully understand the infection history of surgical patients before operation for occupational protection	196 (72)	71 (26.1)	3 (1.1)	2 (0.7)	0 (0)
A2: wearing protective equipment during orthopedic surgery is a bit of a fuss	58 (21.3)	59 (21.7)	65 (23.9)	61 (22.4)	29 (10.7)
A3: It is important to wear N95 masks when using electric drills, grinding drills, high-speed sawing and pulsed irrigation in orthopedic surgery, which are prone to producing aerosols	51 (18.8)	77 (28.3)	106 (39.0)	32 (11.8)	6 (2.2)
A4: When transferring or using a power tool, the handle must be locked or adjusted to the closed state	31 (11.4)	43 (15.8)	42 (15.4)	113 (41.5)	43 (15.8)
A5: A disposable medical surgical gown should be worn to prevent fluid penetration in orthopedic surgeries	87 (32.0)	96 (35.3)	67 (24.6)	22 (8.1)	0 (0)
A6: It is not necessary to wear double gloves during orthopedic surgery if patients are not suffering from infectious diseases	40 (14.7)	60 (22.1)	65 (23.9)	76 (27.9)	31 (11.4)
A7: On the operating table, power instruments including sharp instruments should be properly placed	173 (63.6)	92 (33.8)	7 (2.6)	0 (0)	0 (0)
A8: Ensure that high-risk power tools or sharp instruments pass through a transition area, and prohibit two people from touching the same sharp instrument at the same time	124 (45.6)	97 (35.7)	50 (18.4)	1 (0.4)	0 (0)
A9: The electric drill should be equipped with a protective sleeve to prevent blood splashing and bit breakage	115 (42.3)	114 (41.9)	42 (15.4)	1 (0.4)	0 (0)
A10: The protective caps of sharp instrument tips such as Kirschner wires, drills and conical opening instruments should not be removed at will before use, and the removal and separation should be carried out with the help of instruments	74 (27.2)	143 (52.6)	54 (19.9)	1 (0.4)	0 (0)
A11: To reduce the risk of eye and face exposure, it is important to use goggles and a face mask	53 (19.5)	132 (48.5)	79 (29.0)	8 (2.9)	0 (0)
A12: Sharps such as Kirschner needles and pendulum saw blades should be put into special sharps boxes in a timely manner after use	111 (40.8)	117 (43.0)	43 (15.8)	1 (0.4)	0 (0)
A13: Hearing protection for medical staff in orthopedic surgery is of little significance	31 (11.4)	43 (15.8)	42 (15.4)	113 (41.5)	43 (15.8)
A14: It is important to participate in the study and training of occupational protection knowledge	133 (48.9)	111 (40.8)	27 (9.9)	1 (0.4)	0 (0)
A15: The policy guarantee of occupational protection for medical staff in the operating room is very important	139 (51.1)	103 (37.9)	29 (10.7)	1 (0.4)	0 (0)

The median Practice score was 48 (42, 55), with a minimum score of 28 and a maximum score of 71. On the basis of a median score of 48 as the cutoff, 53.7% (146/272) of the participants had good performance in terms of their Practice score, while 46.3% (126/272) had poor performance. In clinical practice, orthopedic theatre nurses had good compliance with “designating a separate area for power equipment” and “retrieving sharp objects in a timely manner” (the often/always implementation rates were approximately 73% and 68%, respectively). However, their behaviors regarding noise protection and facial protection are weak. Nearly 65% “occasionally or never” wore earplugs when high-noise power tools were used, and approximately 51% did not consistently wear goggles or masks; (details are provided in Table 4). It is suggested that hearing and facial protection are the key shortcomings of the next behavioral intervention in the orthopedic theatre.

**Table 4:** Practice dimension score distribution.

	Always (5)	Often (4)	Sometimes (3)	Occasionally (2)	Never (1)
P1: Before orthopedic surgery, I review the patient's medical history and infectious disease series	48 (17.6)	148 (54.4)	70 (25.7)	6 (2.2)	0 (0)
P2: In order to prevent bone debris, blood, and body fluids from splashing, I will wear appropriate personal protective equipment during the operation	53 (19.5)	42 (15.4)	69 (25.4)	37 (13.6)	71 (26.1)
P3: I wear an N95 mask when participating in surgeries that are prone to producing aerosols, such as joint replacement and screw internal fixation	21 (7.7)	55 (20.2)	87 (32.0)	58 (21.3)	51 (18.8)
P4: I use goggles or a face mask when participating in orthopedic procedures	38 (14.0)	28 (10.3)	73 (26.8)	43 (15.8)	90 (33.1)
P5: For procedures that involve substantial spattering of blood or body fluids or the need for large irrigations, I wear a disposable gown that protects against fluid penetration	31 (11.4)	52 (19.1)	108 (39.7)	54 (19.9)	27 (9.9)
P6: As an instrument nurse, I wear double gloves regardless of whether the blood of orthopedic patients is infectious or not	42 (15.4)	72 (26.6)	72 (26.5)	73 (26.8)	13 (4.8)
P7: I always check the integrity of the openers, grinding drills, power drills and other equipment	74 (27.2)	62 (22.8)	88 (32.4)	48 (17.6)	0
P8: I will set up a special area on the operating table for power instruments, Kirschner wires, sharp instruments, etc.	48 (17.6)	152 (55.9)	69 (25.4)	3 (1.1)	0
P9: I will hand the power device directly to the surgeon because of time constraints or the surgeon's urging	53 (19.5)	45 (16.5)	61 (22.4)	90 (33.1)	23 (8.5)
P10: When using or passing an orthopedic drill, I install the drill sleeve in advance	72 (26.5)	90 (33.1)	44 (16.2)	46 (16.9)	72 (7.4)
P11: I always use tools to remove the protective cap on the tip of orthopedic internal fixation sharps	91 (33.5)	72 (26.5)	49 (18.0)	50 (18.4)	10 (3.7)
P12: When I pass or use a power tool, I lock the power switch in a timely manner	57 (21.0)	87 (32.0)	84 (30.9)	37 (13.6)	7 (2.6)
P13: I will put the used sharps into a special sharps box in a timely manner	58 (21.3)	127 (46.7)	79 (29.0)	8 (2.9)	0
P14: I don't have much time to learn about occupational protection in the operating room	19 (7.0)	64 (23.5)	76 (27.9)	93 (34.2)	20 (7.4)
P15: When using noisy power tools, I wear earplugs or earmuffs to protect my hearing	23 (8.5)	21 (7.7)	52 (19.1)	39 (14.3)	137 (50.4)

### Influencing factors of KAP toward occupational protection when OPTs are used

As shown in Table 5, significant positive correlations were observed among knowledge, attitude and practice (all P < 0.001). The strongest correlation was found between knowledge and attitude (r = 0.423), followed by knowledge–practice (r = 0.343) and attitude–practice (r = 0.335), indicating that higher knowledge scores were associated with more positive attitudes and better compliance.

**Table 5:** Correlation analysis.

	Knowledge	Attitude	Practice
Knowledge	1	/	/
Attitude	0.423^*^	1	/
Practice	0.343^*^	0.335^*^	1

* P < 0.001.

Multivariable logistic regression analyses were performed to assess the factors potentially associated with KAP scores (Table 6). Nurses with a bachelor’s degree, those with a senior professional title, and those who regularly participate in training have significantly higher knowledge scores; among them, the effect is strongest when the training frequency is no more than once every 6 months (aOR = 10.1).

**Table 6:** Multivariable statistical analysis.

Characteristics	Knowledge Dimension ^a^ (Cutoff: ≥ 47/ < 47^d^)		Attitude Dimension ^b^ (Cutoff: ≥ 59/ < 59^d^)		Practice dimension ^c^ (Cutoff: ≥ 48/ < 48^d^)	
	aOR (95% CI)^e^	P	aOR (95% CI)^e^	P	aOR (95% CI)^e^	P
**Education level**						
College degree	ref.					
Bachelor’s degree	7.040 (3.204,15.466)	**<0.001**				
**Professional title**						
Junior	ref.		ref.			
Intermediate	1.782 (0.938,3.386)	0.077	1.089 (0.640,1.854)	0.753		
Senior	5.216 (1.228,22.155)	**0.025**	5.226 (1.409,19.387)	**0.013**		
**Whether actively consulted the latest professional protection knowledge**						
No	ref.				ref.	
Yes	2.652 (1.426,4.931)	**0.002**			2.962 (1.686,5.201)	**0.001**
**Occupational protection training frequency**						
Never	ref.					
Every 6–12 months	6.550 (3.262,13.153)	**<0.001**				
Less than 6 months	10.085 (4.146,24.533)	**<0.001**				
**History of occupational exposure**			ref.			
No			2.770 (1.484,5.169)	**0.001**		
Yes						
**Knowledge scores**			ref.			
<47			2.663 (1.581,4.448)	**<0.001**	ref.	
≥47					2.255 (1.294,3.930)	**0.004**
**Attitude scores**						
<59					ref.	
≥59					3.683 (2.106,6.441)	**<0.001**

^a^Education level, professional title, whether the latest professional protection knowledge was actively consulted and occupational protection training frequency were included in the Knowledge Dimension model.

^b^Professional title, history of occupational exposure, and knowledge scores were included in the Attitude Dimension model.

^c^Whether the latest professional protection knowledge was actively consulted, knowledge scores, and attitude scores were included in the Attitude Dimension model.

^d^Using the median values of the Knowledge, Attitude and Practice scores as the dividing criterion.

^e^aOR, adjusted odds ratio.

Nurses with a senior professional title, a high knowledge score, and prior occupational exposure have significantly higher scores for their occupational protection attitude; among them, senior professional title and prior exposure had the strongest positive effects (aOR=5.2 and 2.8, respectively). Nurses with higher professional titles are more likely to recognize the importance of occupational protection, and participants who have experienced occupational exposure can more profoundly understand the importance of occupational protection.

Three important factors contributing to the improvement in practical scores were positive attitude, active exploration of new knowledge and a high level of knowledge. The most significant effect was positive attitude (aOR=3.7). To effectively improve occupational protective behaviors, attitude transformation is crucial. Additionally, updating knowledge and fostering motivation for active learning are necessary.

## Discussion

Operating room nurses are categorized into a high-risk group for occupational exposure [2]. Especially during orthopedic surgery, when operating power instruments are used, operating room nurses face a variety of high-risk factors [15–17]. This survey revealed that the median score for knowledge accounted for approximately 62.7% (47/75) of the total score, and the 75th percentile score was only 52.7 points, indicating that at least 50% of the participants scored ≤ 47 points, which was less than two-thirds of the total score. The participants in the top 25% of the best results (the 75th percentile) obtained only 52.7 points, just above 70%. The overall level of knowledge is relatively low, which suggests the existence of systematic knowledge gaps. The median for attitude is approximately 78.7% (59/75), and the 25th percentile score is 54 points; some people have achieved a full score of 75 points, indicating that the overall attitude of the population is positive. The attitude dimension has the best performance. The median practice score is approximately 64% (48/75), which is close to the knowledge score. However, the maximum score is 71, suggesting that “High attitude” has not yet fully translated into “High behavior”, and there may be execution obstacles. Therefore, the behavioral dimension is at a moderately low level. On the basis of the conclusions of the above research, we can draw the following conclusions. Nurses in the orthopedic operating room had a positive attitude toward occupational protection for the use of power tools, but their knowledge was insufficient, and their practice behavior needs to be improved.

Knowledge, attitude and practice were moderately positively correlated with each other (r = 0.34–0.42; all p < 0.001), suggesting that the trend of “the more one knows, the higher one’s recognition and the better one’s performance” holds true among orthopedic operating room nurses. However, there is still considerable room for improvement in the correlation. Education and training can enhance knowledge while also indirectly driving attitude change and behavior improvement.

Moralejo et al. [11] conducted a study involving 673 participants (with 3 studies conducted in Asia, 2 in Europe, 2 in North America, and 1 in Australia), and the results indicated that although the organization widely adopts standard preventive measures, there are still gaps in the implementation of these measures by medical staff, especially in terms of facial protection. A study [18] conducted in Canada revealed that the rates of double gloving leave room for improvement. Among the 282 respondents who reported having worn double-layer gloves before, 188 people (66.7%) (accounting for 52.1% of all respondents in the survey) stated that they did this frequently. A total of 300/353 respondents (85.0%) reported using eye protection routinely in the operating room. Research conducted in the Economic Community of West African States by Ridge et al. [19] revealed that 57.7% of medical staff reported that they did not receive personal protective equipment on a regular basis; moreover, 62% of nurses used double-layered gloves at some point. In a study by Roberts et al. [20] in NHANES (National Health and Nutrition Examination Surveys), participants wore ear protection devices when it was mandatory to use them. The present results [21] revealed that orthopedic surgeons are regularly exposed to damaging noise levels (>85 dB), putting them at risk of permanent hearing loss. However, very few data on hearing protection when power tools are used in the operating room have been reported.

Through this study, we identified several issues related to the occupational protection of nurses in the orthopedic surgery department [22–24]. (1) “Double low” facial protection: low awareness rate and low wearing rate, which means that blood-borne pathogens can infect human body through the conjunctiva and the newly shaved cheeks and neck area. When HIV/HBV infection rates are < 1%, a single exposure is low risk, but the cumulative risk increases over a long period of time. (2) The “double-glove” controversy highlights the misunderstanding of “risk calculation”: nurses equated “no patient infection record” with zero risk, ignoring the window period, concealment of medical history and the fact that the gloves had an 18% perforation rate during surgery [25]. (3) In addition, the noise level of power tools is ≥ 145 dB, which is more than 2 times the OSHA (Occupational Safety & Health Administration) exposure limit, but only one-third of personnel are aware of it and one-third wear hearing protection; long-term exposure leads to high-frequency hearing loss, and early damage is irreversible [26].

**This research has certain guiding significance for nursing and occupational safety practices.**

(1) This study aims to contribute to the improvement of policies, incorporating facial blood exposure protection and hearing protection for noise levels ≥ 85 dB into the occupational protection guidelines and supplementing the standards for facial and noise protection for nurses in orthopedic operating rooms.
(2) Data gaps: The WHO/ILO (International Labor Organization) Occupational Safety Database lacked an entry for an orthopedic subspecialty. This study calls for the establishment of “surgical species-tool-exposure” three-dimensional coding to fill this gap.
(3) Training centered on high-frequency and institutionalized training, with highly educated and senior-qualified nurses as exemplary leaders, through onsite peer teaching, continuous knowledge updates, and professional protection education for operating room nurses can be deepened.
(4) Improving personal protective equipment (PPE): In this study, many participants indicated that wearing double gloves, face shields, or hearing protective earplugs could affect surgical procedures. Existing PPE is not completely suitable for use in orthopedic and trauma surgeries; therefore, convenient and practical protective equipment is urgently needed.

### Limitations

Through a multicenter cross-sectional survey conducted in 8 tertiary hospitals in Shanxi Province, this study clarified the knowledge, attitudes, and practices regarding occupational protection among orthopedic theatre nurses using orthopedic power tools. However, this study has several limitations. Owing to sample size limitations, the sample of this study may not adequately represent all orthopedic operating room nurses in Shanxi Province. The sample size should be increased for further research. Second, this questionnaire was completed by the participants themselves, which may have led to some subjective bias. Therefore, the assessment of their knowledge, attitude and behavior levels might be either overestimated or underestimated. Third, although the present findings support the preliminary validity of the scale, there are a few items whose standardized load values are not very satisfactory. In future research, some of these items should be integrated or modified on the basis of an expansion of the sample size. In addition, the evidence is based on cross-sectional self-report data; therefore, we recommend future validation against objective safety indicators (e.g., injury records). Finally, future work should establish longitudinal measurement invariance and expand to other geographical and cultural backgrounds.

## Conclusions

This study revealed that the attitude of nurses in the orthopedic operating room was positive toward occupational protection when using power tools; however, their knowledge was insufficient, and their practical behaviors required improvement. Systematic continuing education and awareness of active learning significantly promote knowledge acquisition. Knowledge factors can influence attitude formation, whereas occupational exposure history may strengthen protective awareness through risk perception. Knowledge and attitude eventually translate into behavior, and active learning, as a continuous factor, runs through the entire process. Orthopedic operating room nurses are strongly willing to participate in occupational protection training. The organizational management departments of medical institutions providing policy support and conducting occupational health training regularly could further facilitate the implementation of the occupational protection behaviors of nurses in the orthopedic operating theatre.

Normative guidelines for occupational protection in the workplace of an orthopedic operating theatre and a training and evaluation system should be developed. In addition, strengthening education and training is essential, particularly regarding basic knowledge on the importance of wearing double gloves, protection against aerosols and prevention of noise-induced hearing loss. Additionally, developing and designing occupational protective equipment that is convenient for use in orthopedic surgery is important.

## Supporting information

S1 Table Original data. (XLSX)

S2 Table Questionnaire. (DOCX)

S3 Table Bivariable statistical analysis. (DOCX)

## References

[1] Mengistu DA, Tolera ST, Demmu YM. Worldwide prevalence of occupational exposure to needle stick injury among healthcare workers: a systematic review and meta-analysis. Can J Infect Dis Med Microbiol. 2021;2021:9019534. doi: 10.1155/2021/9019534 33564345 PMC7864758

[2] Association of periOperative Registered Nurses. AORN guidance statement: sharps injury prevention in the perioperative setting. AORN J. 2005;81(3):662, 665–6, 669–71. doi: 10.1016/s0001-2092(06)60449-3 15799514

[3] Cheetham S, Ngo HT, Liira J, Liira H. Education and training for preventing sharps injuries and splash exposures in healthcare workers. Cochrane Database Syst Rev. 2021;4(4):CD012060. doi: 10.1002/14651858.CD012060.pub2 33871067 PMC8094230

[4] Jeyaraman M, Jeyaraman N, Yadav S, Nallakumarasamy A, Iyengar KP, Jain V. Impact of excessive noise generation in orthopaedic operating theatres: a comprehensive review. Cureus. 2024;16(2):e54469. doi: 10.7759/cureus.54469 38510860 PMC10951741

[5] Basso T, Dale H, Langvatn H, Lønne G, Skråmm I, Westberg M, et al. Virus transmission during orthopedic surgery on patients with COVID-19 - a brief narrative review. Acta Orthop. 2020;91(5):534–7. doi: 10.1080/17453674.2020.1764234 32408845 PMC8023894

[6] Alaqeel M, Tanzer M. Improving ergonomics in the operating room for orthopaedic surgeons in order to reduce work-related musculoskeletal injuries. Ann Med Surg (Lond). 2020;56:133–8. doi: 10.1016/j.amsu.2020.06.020 32637088 PMC7327029

[7] Ferrario MM, Veronesi G, Borchini R, Cavicchiolo M, Dashi O, Dalla Gasperina D, et al. Time trends of percutaneous injuries in hospital nurses: evidence of the interference between effects of adoption of safety devices and organizational factors. Int J Environ Res Public Health. 2021;18(8):4371. doi: 10.3390/ijerph18084371 33924104 PMC8074301

[8] Ryu RC, Behrens PH, Malik AT, Lester JD, Ahmad CS. Are we putting ourselves in danger? Occupational hazards and job safety for orthopaedic surgeons. J Orthop. 2021;24:96–101. doi: 10.1016/j.jor.2021.02.023 33716416 PMC7920798

[9] Geevarughese NM, Haq R-U. Aerosol generating procedures in orthopaedics and recommended protective gear. J Clin Orthop Trauma. 2021;12(1):40–2. doi: 10.1016/j.jcot.2020.08.019 32863676 PMC7446649

[10] Endo S, Kanemitsu K, Ishii H, Narita M, Nemoto T, Yaginuma G, et al. Risk of facial splashes in four major surgical specialties in a multicentre study. J Hosp Infect. 2007;67(1):56–61. doi: 10.1016/j.jhin.2007.05.020 17669549

[11] Moralejo D, El Dib R, Prata RA, Barretti P, Corrêa I. Improving adherence to standard precautions for the control of health care-associated infections. Cochrane Database Syst Rev. 2018;2(2):CD010768. doi: 10.1002/14651858.CD010768.pub2 29481693 PMC6491237

[12] Nogler M, Lass-Flörl C, Wimmer C, Bach C, Kaufmann C, Ogon M. Aerosols produced by high-speed cutters in cervical spine surgery: extent of environmental contamination. Eur Spine J. 2001;10(4):274–7. doi: 10.1007/s005860100310 11563611 PMC3611509

[13] Lester JD, Hsu S, Ahmad CS. Occupational hazards facing orthopedic surgeons. Am J Orthop (Belle Mead NJ). 2012;41(3):132–9. 22530210

[14] Palmer JS, Wilson C, Fraig H, Wilson D, Garrett S. Hearing Evaluation of ARthroplasty Surgeons: results from the HEARS study. Ann R Coll Surg Engl. 2021;103(9):673–7. doi: 10.1308/rcsann.2021.0050 33956515 PMC10334960

[15] Hafeez H, Abdullah MI, Riaz A, Shafique I. Prevention of occupational injuries and accidents: a social capital perspective. Nurs Inq. 2020;27(4):e12354. doi: 10.1111/nin.12354 32406124

[16] Sobti A, Fathi M, Mokhtar MA, Mahana K, Rashid MS, Polyzois I, et al. Aerosol generating procedures in trauma and orthopaedics in the era of the Covid-19 pandemic; What do we know? Surgeon. 2021;19(2):e42–8. doi: 10.1016/j.surge.2020.08.001 32883580 PMC7425761

[17] Kugelman D, Weppler CG, Warren CF, Lajam CM. Occupational hazards of orthopedic surgery exposures: infection, smoke, and noise. J Arthroplasty. 2022;37(8):1470–3. doi: 10.1016/j.arth.2022.03.034 35304300

[18] Lipson ME, Deardon R, Switzer NJ, de Gara C, Ball CG, Grondin SC. Practice and attitudes regarding double gloving among staff surgeons and surgical trainees. Can J Surg. 2018;61(4):244–50. doi: 10.1503/cjs.013616 30067182 PMC6066380

[19] Ridge LJ, Dickson VV, Stimpfel AW. The occupational health of nurses in the economic community of West African states: a review of the literature. Workplace Health Saf. 2019;67(11):554–64. doi: 10.1177/2165079919859383 31364508 PMC8312320

[20] Roberts B, Smith S, Vahora M, Miller E. Self-reported occupational noise exposure and hearing protection device use among NHANES participants and the risk of hearing loss. J Occup Environ Hyg. 2024;21(9):623–8. doi: 10.1080/15459624.2024.2371904 39042873

[21] Kwan SA, Lynch JC, DeFrance M, Ciesielka K-A, Rivlin M, Daniel JN. Risk of noise-induced hearing loss for orthopaedic surgeons. J Bone Joint Surg Am. 2022;104(23):2053–8. doi: 10.2106/JBJS.22.00582 36170382

[22] Landford WN, Ngaage LM, Lee E, Rasko Y, Yang R, Slezak S, et al. Occupational exposures in the operating room: Are surgeons well-equipped? PLoS One. 2021;16(7):e0253785. doi: 10.1371/journal.pone.0253785 34214125 PMC8253435

[23] Persaud E, Parker GB, Mitchell AH. Blood and body fluid exposure among healthcare workers and personal protective equipment usage in the United States. Workplace Health Saf. 2023;71(9):429–35. doi: 10.1177/21650799231163132 37232173

[24] Yakkanti RR, Sedani AB, Syros A, Aiyer AA, D’Apuzzo MR, Hernandez VH. Prevalence and spectrum of occupational injury among orthopaedic surgeons. JBJS Open Access. 2023;8(1). doi: 10.2106/jbjs.oa.22.00083 PMC9886518 36733707

[25] Santol J, Willegger M, Hanreich C, Albrecht L, Lisy M, Hajdu S, et al. Surgical glove perforation during intramedullary nailing of intertrochanteric fractures. Sci Rep. 2025;15(1):1203. doi: 10.1038/s41598-024-84994-w 39774284 PMC11707250

[26] Pan DW, Choi JS, Hokan A, Doherty JK. Trends in hearing protection use with occupational noise exposure in the United States 1999 to 2016. Otol Neurotol. 2022;43(1):e14–22. doi: 10.1097/MAO.0000000000003343 34510117

