# Supplementary material for: Knowledge, attitudes, and practices (KAP) regarding occupational protection among orthopedic theatre nurses using orthopedic power tools (OPTs): A cross-sectional study
Source: PLoS One. 2026 May 15;21(5):e0349690. doi: 10.1371/journal.pone.0349690 (PMC13178885; doi:10.1371/journal.pone.0349690)
Supplement: S2 Table — (DOCX) [file pone.0349690.s002.docx]

This section is a questionnaire about the occupational protection knowledge of orthopedic surgery power tools for nurses in the orthopedic surgery room. It consists of 15 items and a 5-point Likert scale was used to score the items. Please answer based on your own situation, truthfully and patiently. Thank you for your cooperation. (5- Completely clear, 4- Basically clear, 3-Partly clear, 2-Not very clear, 1-Not clear at all)

**Table 1** **The knowledge of occupational protection about orthopedic power tools**

|  | **Completely clear（5）** | **Basically clear（4）** | **Partly clear（3）** | **Not very clear（2）** | **Not clear at all（1）** |
| --- | --- | --- | --- | --- | --- |
| K1: Patients should be screened for anti-HIV, anti-HCV, HBsAg and syphilis antibodies before operation |  |  |  |  |  |
| K2: In close contact with blood and irrigating fluid, power instruments in orthopedic surgery will atomize and form aerosols |  |  |  |  |  |
| K3:The aerosol cloud generated during orthopedic surgery can be contaminated by microorganisms such as viruses, bacteria and fungi |  |  |  |  |  |
| K4: I am aware of the high-risk procedures for aerosol generation in orthopedic surgery |  |  |  |  |  |
| K5: Conventional surgical masks do not protect against procedures that produce high risk aerosols (N95 or FFP2 masks are preferred) |  |  |  |  |  |
| K6：In order to prevent bone debris, blood and body fluids from splashing, the personnel involved in the operation should wear appropriate personal protective equipment |  |  |  |  |  |
| K7: Use goggles to protect the conjunctiva from contamination |  |  |  |  |  |
| K8: To effectively protect the newly shaved cheeks and neck area, male health care providers are recommended to wear a face mask |  |  |  |  |  |
| K9: The electric drill should be equipped with a protective sleeve to prevent blood splashing and bit breakage |  |  |  |  |  |
| K10: The use of power equipment is easy to wear the skin of the hand, and wearing double gloves can reduce the risk of penetration of the inner gloves by 6 times |  |  |  |  |  |
| K11: Ensure that high-risk power instruments or sharp tools pass through a transition area |  |  |  |  |  |
| K12: Use instruments or auxiliary tools to install or remove power tools |  |  |  |  |  |
| K13: Operations such as electric bone drills, cutting saws, and hammer hammering can generate up to 145 dB of noise |  |  |  |  |  |
| K14: When the intraoperative noise value is greater than 85dB, hearing protection is recommended for medical staff |  |  |  |  |  |
| K15: I know the principles of management and reporting procedures after occupational exposure |  |  |  |  |  |

This section is a questionnaire about the occupational protection attitude of orthopedic surgery power tools for nurses in the orthopedic surgery room. It consists of 15 items and a 5-point Likert scale was used to score the items. Thank you for your cooperation.

(5- Completely agree, 4- Basically agree, 3- Moderate, 2- Disagree, 1- Completely disagree)

**Table 2 The attitude of occupational protection about orthopedic power tools**

|  | **Completely agree（5）** | **Basically agree（4）** | **Moderate（3）** | **Disagree（2）** | **Completely disagree（1）** |
| --- | --- | --- | --- | --- | --- |
| A1:It is important to fully understand the infection history of surgical patients before operation for occupational protection |  |  |  |  |  |
| A2: wearing protective equipment during orthopedic surgery was a bit of a fuss |  |  |  |  |  |
| A3: It is important to wear N95 masks when using electric drills, grinding drills, high-speed sawing and pulsed irrigation in orthopedic surgery, which are prone to produce aerosols |  |  |  |  |  |
| A4: When transferring or using the power tool, the handle must be locked or adjusted to the closed state |  |  |  |  |  |
| A5: Disposable medical surgical gown should be worn to prevent fluid penetration in orthopedic surgeries |  |  |  |  |  |
| A6: It is not necessary to wear double gloves during orthopedic surgery if patients are not suffering from infectious diseases |  |  |  |  |  |
| A7: On the operating table, power instruments including sharp instruments should be properly placed |  |  |  |  |  |
| A8: Ensure that high-risk power tools or sharp instruments pass through a transition area, and prohibit two people from touching the same sharp instrument at the same time |  |  |  |  |  |
| A9: The electric drill should be equipped with a protective sleeve to prevent blood splashing and bit breakage |  |  |  |  |  |
| A10: The protective caps of sharp instrument tips such as Kirschner wires, drills and conical opening instruments should not be removed at will before use, and the removal and separation should be carried out with the help of instruments |  |  |  |  |  |
| A11: To reduce the risk of eye and face exposure, it is important to use goggles or a face mask |  |  |  |  |  |
| A12: Sharps such as Kirschner needles and pendulum saw blades should be put into special sharps boxes in time after use |  |  |  |  |  |
| A13: Hearing protection for medical staff in orthopedic surgery is of little significance |  |  |  |  |  |
| A14: It is important to participate in the study and training of occupational protection knowledge |  |  |  |  |  |
| A15: The policy guarantee of occupational protection for medical staff in operating room is very important |  |  |  |  |  |

This section is a questionnaire about the occupational protection practice of orthopedic surgery power tools for nurses in the orthopedic surgery room. It consists of 15 items and a 5-point Likert scale was used to score the items. Thank you for your cooperation.

(5- Always, 4- Often, 3- Sometimes, 2- Occasionally, 1- Never)

**Table 3 The practice of occupational protection about orthopedic power tools**

|  | **Always(5)** | **Often(4)** | **Sometimes(3)** | **Occasionally(2)** | **Never(1)** |
| --- | --- | --- | --- | --- | --- |
| P1:Before orthopedic surgery, I review the patient's medical history and infectious disease series |  |  |  |  |  |
| P2: In order to prevent bone debris, blood, and body fluids from splashing, I will wear appropriate personal protective equipment during the operation |  |  |  |  |  |
| P3: I wear an N95 mask when participating in surgeries that are prone to producing aerosols, such as joint replacement and screw internal fixation |  |  |  |  |  |
| P4: I use goggles or a face mask when participating in orthopedic procedures |  |  |  |  |  |
| P5: For procedures that involve substantial spattering of blood or body fluids or the need for large irrigations, I wear a disposable gown that protects against fluid penetration |  |  |  |  |  |
| P6: As an instrument nurse, I wear double gloves regardless of whether the blood of orthopedic patients is infectious or not |  |  |  |  |  |
| P7: I always check the integrity of the openers, grinding drills, power drills and other equipment |  |  |  |  |  |
| P8: I will set up a special area on the operating table for power instruments, Kirschner wires, sharp instruments, et al |  |  |  |  |  |
| P9:I will hand the power device directly to the surgeon because of time constraints or the surgeon's urging |  |  |  |  |  |
| P10: When using or passing an orthopedic drill, I install the drill sleeve in advance |  |  |  |  |  |
| P11: I always use tools to remove the protective cap on the tip of orthopedic internal fixation sharps |  |  |  |  |  |
| P12: When I pass or use the power tool, I lock the power switch in time |  |  |  |  |  |
| P13: I will put the used sharps into the special sharps box in time |  |  |  |  |  |
| P14: I don't have much time to learn about occupational protection in the operating room |  |  |  |  |  |
| P15: When using noisy power tools, I wear earplugs or earmuffs to protect my hearing |  |  |  |  |  |
