# Supplementary material for: Knowledge, attitudes, and practices (KAP) regarding occupational protection among orthopedic theatre nurses using orthopedic power tools (OPTs): A cross-sectional study
Source: PLoS One. 2026 May 15;21(5):e0349690. doi: 10.1371/journal.pone.0349690 (PMC13178885; doi:10.1371/journal.pone.0349690)
Supplement: S3 Table — (DOCX) [file pone.0349690.s003.docx]

**Supplementary Table. Bivariable Statistical analysis.**

| Characteristics | Knowledge Dimension  (Cutoff:≥47/<47^a^) | | Attitude Dimension  (Cutoff:≥59/<59^a^) | | Practice dimension  (Cutoff:≥48/<48^a^) | |
| --- | --- | --- | --- | --- | --- | --- |
|  | OR(95%CI)^b^ | P | OR(95%CI)^b^ | P | OR(95%CI)^b^ | P |
| **Gender** |  |  |  |  |  |  |
| Male | ref. |  | ref. |  | ref. |  |
| Female | 0.785(0.439,1.405) | 0.416 | 0.990(0.555,1.766) | 0.973 | 0.972(0.545,1.733) | 0.922 |
| **Education level** |  |  |  |  |  |  |
| College degree | ref. |  | ref. |  | ref. |  |
| Bachelor degree | 6.898(3.532,12.668) | **<0.001** | 2.414(1.385,4.209) | **0.002** | 2.017(1.164,3.496) | **0.012** |
| **Years employed in operation room** |  |  |  |  |  |  |
| <5 | ref. |  | ref. |  | ref. |  |
| ≥5 to <10 | 1.708(0.805,3.621) | 0.163 | 1.050(0.502,2.198) | 0.897 | 2.108(0.979,4.537) | 0.057 |
| ≥10 | 2.109(1.036,4.294） | **0.040** | 1.459(0.726,2.932) | 0.288 | 2.927(1.413,6.064) | **0.004** |
| **Professional title** |  |  |  |  |  |  |
| Junior | ref. |  | ref. |  | ref. |  |
| Intermediate | 1.787(1.082,2.949) | **0.023** | 1.177(0.716,1.933) | 0.521 | 1.381(0.839,2.271) | 0.204 |
| Senior | 8.406(2.368,29.841) | **0.001** | 6.534(1.842,23.175) | **0.004** | 7.178(2.023,25.461) | **0.002** |
| **Occupational protection training willingness** |  |  |  |  |  |  |
| No | ref. |  | ref. |  | ref. |  |
| Yes | 2.481(1.115,5.524) | **0.026** | 1.033(0.483,2.209) | 0.934 | 0.873(0.406,1.876) | 0.728 |
| **occupational protection training Frequency** |  |  |  |  |  |  |
| Never | ref. |  | ref. |  | ref. |  |
| every 6-12 months | 7.778(4.156,14.557) | **<0.001** | 2.069(1.179,3.628) | **0.011** | 2.199(1.253,3.862) | **0.006** |
| less than 6 months | 14.159(6.472,32.656) | **<0.001** | 3.462(1.768,6.776) | **<0.001** | 4.022(2.023,7.960) | **<0.001** |
| **whether actively consulted the latest professional protection knowledge** |  |  |  |  |  |  |
| No | ref. |  | ref. |  | ref. |  |
| Yes | 3.313(2.011,5.458) | **<0.001** | 1.012(0.628,1.631) | 0.961 | 3.123(1.898,5.137) | **<0.001** |
| **History of occupational exposure** |  |  |  |  |  |  |
| No | ref. |  | ref. |  | ref. |  |
| Yes | 1.754(0.998,3.084) | 0.051 | 2.789(1.536,5.066) | **0.001** | 1.432(0.819,2.501) | 0.207 |

^a^ Using the median value of the Knowledge, Attitude and Practice scores as the dividing criterion respectively

^b^ OR, odds ratio; 95%CI, 95% confidence interval
